# Associating the old with the new

**DOI:** 10.7554/eLife.80030

**Published:** 2022-06-15

**Authors:** Chuqi Liu, Gui Xue

**Affiliations:** 1 State Key Laboratory of Cognitive Neuroscience and Learning and the IDG/McGovern Institute for Brain Research, Beijing Normal University Beijing China

**Keywords:** memory reactivation, semantic memory, retroactive interference, retroactive facilitation, memory integration, memory, Human

## Abstract

New memories can strengthen old memories if the recent and past experience contain elements that are semantically related.

**Related research article** Antony J, Romero A, Vierra A, Luenser R, Hawkins R, Bennion K. 2022. Semantic relatedness retroactively boosts memory and promotes memory interdependence across episodes. *eLife* **11**:e72519. doi: 10.7554/eLife.72519.

Unlike the information stored in a computer disk or a digital camera, human memories are tightly connected and interact strongly with one another. For example, seeing a snake in the zoo may remind you of a previous encounter with a snake in the wild, or other dangerous experiences such as being chased by a dog (Figure 1A). But what are the consequences of spontaneously reviving these old memories? Does the new memory interfere and weaken the old memory (also known as retroactive interference), or does remembering the previous experience make the old memory stronger (retroactive facilitation)?

**Figure 1.**
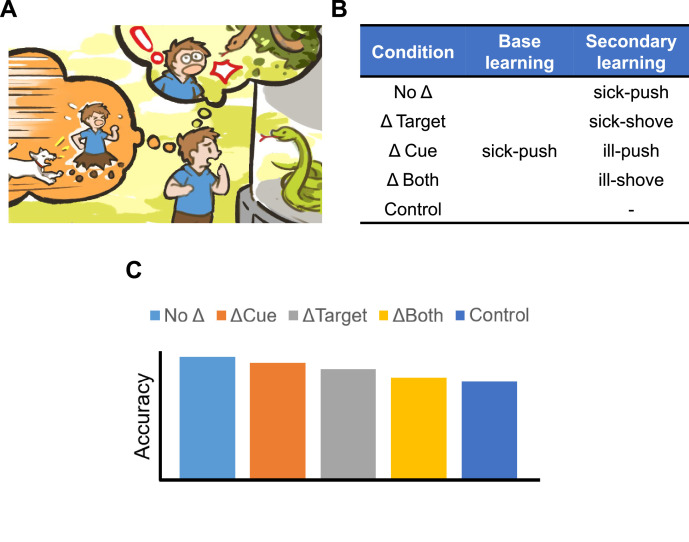
Testing the interaction between semantically related memories. (**A**) Seeing a snake in the zoo may cause you to recall an old related memory, such as the time you saw a snake in the wild or another dangerous experience, like being chased by a dog. (**B**) To test what happens to old memories when they are re-activated and associated with new experiences, Antony et al. carried out a series of associative learning experiments. First, participants were asked to memorize 45 pairs of words (base learning), comprised of a cue (sick) and target (push). They were then given a list of 36 secondary word pairs to learn which either had the same cues and targets (No Δ condition), a new target (Δ Target), a new cue (Δ Cue), or a new cue and a new target (Δ Both), which were semantically related to the words in the base pair. For example, the base pair ‘sick-push’ could lead to the secondary word pairs ‘sick-shove’ or ‘ill-push’. No secondary pairs were created for the control group. (**C**) The schematic depicts how each condition impacted the participants’ memory of the base pairs five minutes or 48 hours after the learning period. All three conditions in which the words in the secondary list were changed to semantically related words improved participants’ memory of the original list compared to the control (dark blue bar), with the Δ Cue condition (orange bar) causing the largest effect and the Δ Both (yellow bar) the smallest.

Image credit: Panel A created by Saeoy; Panel C is based on data from Antony et al., 2022.

To help answer these questions, researchers have traditionally carried out associative learning experiments. In these studies, participants are asked to memorize a list of cues that are each associated with a target, such as pairs of words. They then learn a second list which contains the same cues paired with new targets, or new cues paired with the original targets. It has been proposed that if the cues or targets in the second list are semantically related to the ones initially provided (meaning they have a similar definition or share a common theme), this can influence the participants’ memory of the original list (Osgood, 1949).

Many studies have tested aspects of this theory, but the results are mixed. Some found that having identical cues led to retroactive interference, but this could be reduced by changing the targets to semantically related words (Dallett, 1962). In contrast, other studies found that having highly related cues (but not targets) led to retroactive facilitation and strengthened the participants’ ability to recall the original list (Hamilton, 1943).

Now, in eLife, James Antony and co-workers (from University of California, Davis, California Polytechnic State University and Princeton University) report a series of experiments that help to address these discrepancies (Antony et al., 2022). Unlike previous studies, the team investigated all the possible outcomes of having semantically related old and new target/cue pairs (ranging from identical to weakly related) using the same experimental framework. The impressive sample size and range of experimental conditions studied holds great potential to clarify the influence semantic relatedness has on old memories.

Participants initially learned 45 unrelated word pairs (known as the base pairs) and then an additional 36 pairs (secondary pairs). The secondary pairs were either identical to the original words (No Δ condition), contained new targets (Δ Target condition), new cues (Δ Cue condition), or new cues and new targets (Δ Both condition; Figure 1B). New cues and targets in the secondary lists either came from a set of human-generated words that have a narrow range of semantic relatedness to the base pairs (narrow set), or a computer-generated set that had a broader range of semantic relatedness (wider set). Participants (800 in total) were then tested on their ability to recall the original base pairs and the relevant secondary list five minutes or 48 hours after the learning period. The memory dependence of each base-second pair duo was subsequently calculated by quantifying the proportion of participants who simultaneously remembered or forgot the corresponding word pairs during the test.

This revealed that having secondary words that are narrowly or widely related to the original base pairs led to strong retroactive facilitation of the old memory, especially for the 48 hour delay condition (Figure 1C). In addition, the Δ Cue condition (where the target was held constant) had an overall more robust effect on facilitating old memories than the Δ Target condition. This demonstrates the asymmetric nature of semantic relatedness, with forward associations often being stronger than backward associations.

Antony et al. found that increasing the semantic relatedness between target words in the base and secondary pair improved retroactive facilitation and memory dependence in the majority of conditions. However, increasing the association between cue words did not have the same effect. Notably, when participants were asked to learn secondary Δ Target pairs made using the wider set, this interfered with their memory of the original list after a short delay, but strengthened it following a long delay. This suggests that retroactive interference is only temporary.

So, why does information that is learned later (i.e. the secondary list) benefit prior memories? One likely mechanism is the ‘recursive reminder hypothesis’ which posits that related old information is spontaneously reactivated and strengthened during new learning (Jacoby et al., 2015; Schlichting et al., 2015). This can be tested by comparing two learning methods: practicing retrieving the target after being provided with its matching cue vs. repeatedly studying the cue-target pairs.

In all the experiments conducted so far, Antony et al. had asked participants to learn the base and secondary pairs using the retrieval method. So, they repeated one of the conditions (narrow set of words, 48 hour delay) with 200 additional participants who were told to study the cues and targets instead. This condition was chosen because it had led to strong retroactive facilitation when both the cue and target were changed, a surprising result given that the base and secondary pairs did not share any overlapping words. In theory, retrieval practice should lead to more retroactive facilitation than re-study as participants are more likely to reactivate the old related memories when practicing retrieving words on the new list (Chan et al., 2006; Ye et al., 2020). However, Antony et al. found that re-study also led to retroactive facilitation but no memory dependence, and this retroactive facilitation effect was increased by amplifying the semantic relatedness of the target words.

Antony et al. set an excellent example of reconciling longstanding debates through comprehensive conditions, rigorous controls, and large sample sizes. Future studies could use neuroimaging to uncover the neural processes underlying the effects detected by Antony et al. and examine how learning changes the way old and new memories are represented in the brain. These findings will help scientists to better understand how different memories interact when they are semantically related to one another.
